# Supplementary material for: Lack of MDA5 delays hematopoietic aging by modulating inflammaging and proteostasis in mice
Source: Nat Commun. 2026 Feb 12;17:1645. doi: 10.1038/s41467-026-69424-x (PMC12905429; doi:10.1038/s41467-026-69424-x)
Supplement: Supplementary file 1 — Supplementary Information [file 41467_2026_69424_MOESM1_ESM.pdf]

Supplementary Information

Lack of MDA5 delays hematopoietic aging by modulating inflammaging and proteostasis in mice

Veronica Bergo[1-3#*], Pavlos Bousounis[1,2#], Giang To Vu[4#], Mélodie Douté[5], Aikaterini Polyzou[1,2,4], Maria-Eleni Lalioti[6], Bogdan B. Grigorash[4], Lyudmila Tsurkan[5], Nicholas Morchel[5], Ward Deboutte[1], Frédéric Brau[4], Thomas Manke[1,7], Sagar[8], Hind Medyouf[9], Dmitry V. Bulavin[4], Nina Cabezas-Wallscheid[6], Marta Derecka[5*], Eirini Trompouki[1,4*]

equal first authors

*Corresponding authors

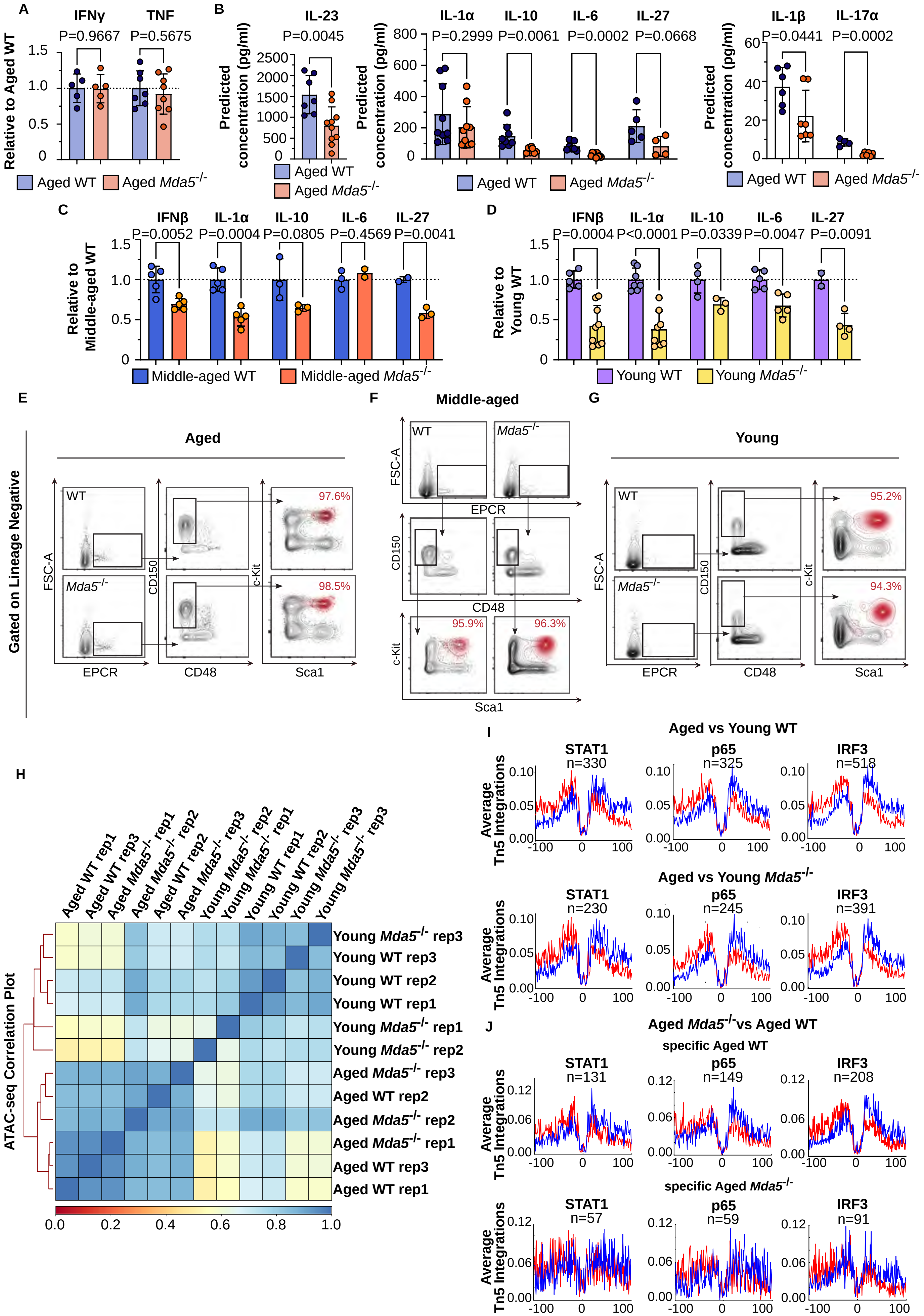

A
IFNγ TNF
P=0.9667 P=0.5675
Relative to Aged WT
Aged WT
Aged Mda5-/-
B
IL-23
P=0.0045
Predicted concentration (pg/ml)
IL-1α IL-10 IL-6 IL-27
P=0.2999 P=0.0061 P=0.0002 P=0.0668
IL-1β IL-17α
P=0.0441 P=0.0002
C
IFNβ IL-1α IL-10 IL-6 IL-27
P=0.0052 P=0.0004 P=0.0805 P=0.4569 P=0.0041
Relative to Middle-aged WT
Middle-aged WT
Middle-aged Mda5-/-
D
IFNβ IL-1α IL-10 IL-6 IL-27
P=0.0004 P<0.0001 P=0.0339 P=0.0047 P=0.0091
Relative to Young WT
Young WT
Young Mda5-/-
E
Aged
Gated on Lineage Negative
WT
Mda5-/-
97.6%
98.5%
FSC-A
CD150
c-Kit
EPCR
CD48
Sca1
F
Middle-aged
95.9%
96.3%
G
Young
95.2%
94.3%
H
ATAC-seq Correlation Plot
Aged WT rep1
Aged WT rep3
Aged Mda5-/- rep1
Aged Mda5-/- rep2
Aged WT rep2
Aged Mda5-/- rep3
Young Mda5-/- rep2
Young Mda5-/- rep1
Young WT rep1
Young WT rep2
Young WT rep3
Young Mda5-/- rep3
0.0 0.2 0.4 0.6 0.8 1.0
I
Aged vs Young WT
STAT1 n=330
p65 n=325
IRF3 n=518
Average Tn5 Integrations
Aged vs Young Mda5-/-
STAT1 n=230
p65 n=245
IRF3 n=391
J
Aged Mda5-/- vs Aged WT
specific Aged WT
STAT1 n=131
p65 n=149
IRF3 n=208
specific Aged Mda5-/-
STAT1 n=57
p65 n=59
IRF3 n=91

Supplementary Figure 1. Reduced inflammation in *Mda5*$^{-/-}$ HSCs and bone marrow serum. A, Fold change of IFNγ (N=5WT and 5 *Mda5*$^{-/-}$) and TNF (N=7 WT and 8 *Mda5*$^{-/-}$) concentrations in the bone marrow serum of aged WT or *Mda5*$^{-/-}$, relative to WT control. *n*=2 independent experiments. Each dot represents one mouse. Data are presented as mean values ± SD. Two-tailed unpaired t-tests. **B,** Concentrations (pg/ml) of inflammatory cytokines in the bone marrow serum of aged WT or *Mda5*$^{-/-}$ mice. N=7 WT and 10 *Mda5*$^{-/-}$ for IL-23, N=9 WT and 9 *Mda5*$^{-/-}$ for IL-1α, N=8 WT and 7 *Mda5*$^{-/-}$ for IL-10, N=7 WT and 9 *Mda5*$^{-/-}$ for IL-6, N=5 WT and 4 *Mda5*$^{-/-}$ for IL-27, N=6 WT and 7 *Mda5*$^{-/-}$ for IL-1β, N=4 WT and 6 *Mda5*$^{-/-}$ for IL-17α) in *n*=2 independent experiment. Each dot represents one mouse. Data are presented as mean values ± SD. Two-tailed unpaired t-tests. **C,** Fold change of diverse cytokine concentrations in bone marrow serum of middle-aged WT or *Mda5*$^{-/-}$, normalized to corresponding WT controls. N=5 WT and 5 *Mda5*$^{-/-}$ for IFNβ and IL-1α, N=3 WT and 3 *Mda5*$^{-/-}$ for IL-10, N=3 WT and 2 *Mda5*$^{-/-}$ for IL-6 and N=2 WT and 3 *Mda5*$^{-/-}$ for IL-23 in *n*=2 independent experiments. Each dot represents one mouse. Data are presented as mean values ± SD. Two-tailed unpaired t-tests. **D,** Fold change of diverse cytokine concentrations in the bone marrow serum of young WT or *Mda5*$^{-/-}$, normalized to the corresponding WT control. N=5 WT and 9 *Mda5*$^{-/-}$ for IFNβ and N=7 WT and N=8 *Mda5*$^{-/-}$ IL-1α, N=4 WT and 3 *Mda5*$^{-/-}$ for IL-10, N=4 WT and 5 *Mda5*$^{-/-}$ for IL-6 and N=2 WT and 4 *Mda5*$^{-/-}$ for IL-27 in *n*=3 independent experiments. Each dot represents one mouse. Data are presented as mean values ± SD. Two-tailed unpaired t-tests. P (IL-1α) =0.000028. **E-G,** Gating strategy for sorting EPCR SLAM HSCs from the bone marrow of aged (**E**), middle-aged (**F**), and young (**G**) WT or *Mda5*$^{-/-}$ mice, and comparison to the standard gating strategy (LSK SLAM: Lineage-Sca+cKit+ CD48-CD150+) (representative graphs are shown). The EPCR SLAM population (in red) is projected on the LSK SLAM gating strategy, and the percentage of EPCR SLAM HSCs included in the LSK SLAM gate is indicated. *n*=8 biologically independent samples in *n*=2 independent experiments. **H**, ATAC-seq Pearson correlation plot for the indicated ATAC-seq samples **I-J**, Average normalized Tn5 insertion profiles neighboring footprinted motifs (p65, IRF3, STAT1) in merged ATAC peaks from aged versus young WT (**I** up) or *Mda5*$^{-/-}$ (**I** down) or aged *Mda5*$^{-/-}$ versus aged WT (**J**). Footprint numbers (n) are shown at the top. Insertions on the forward and reverse DNA strands are represented in red and blue, respectively.

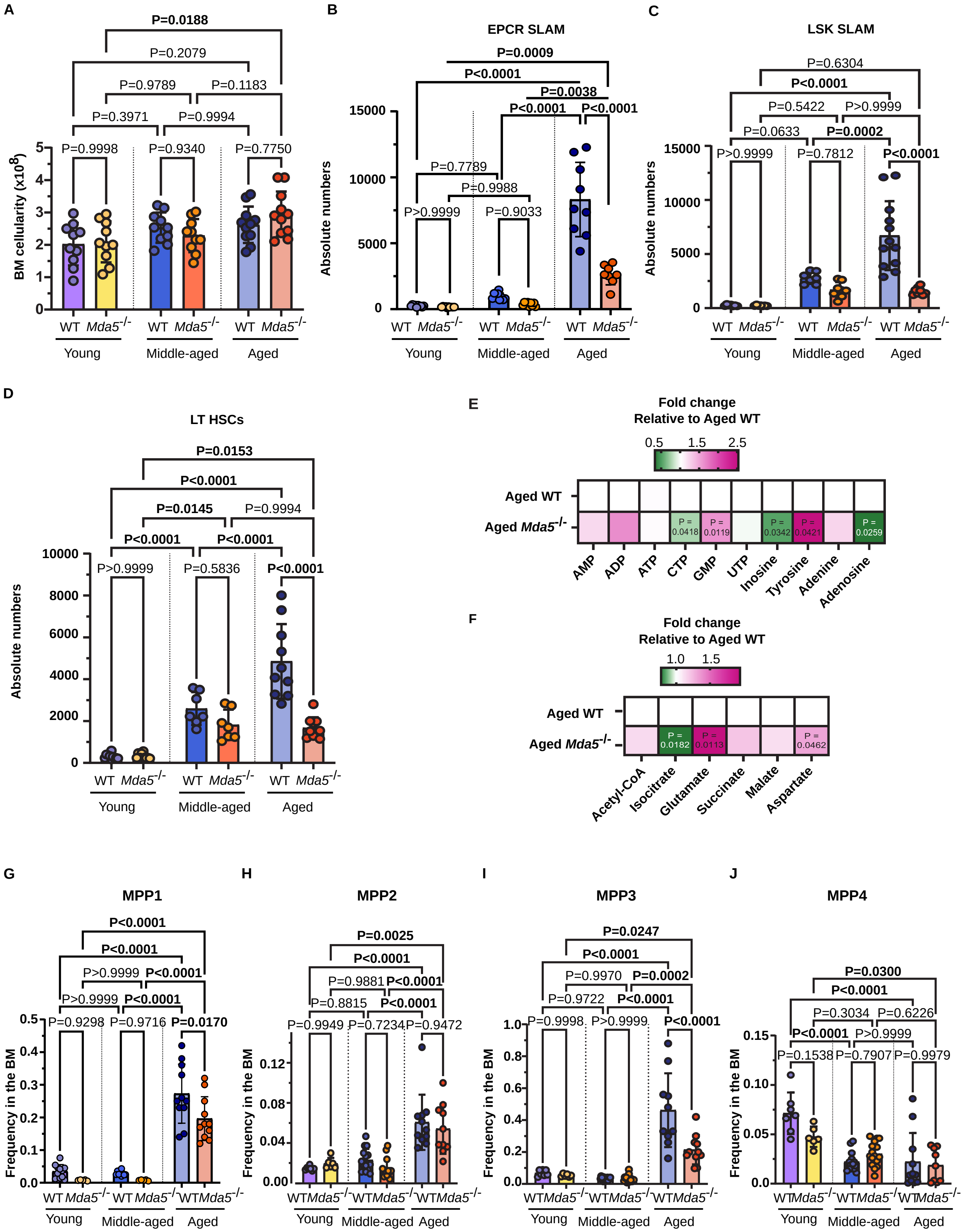

A
P=0.0188
P=0.2079
P=0.9789
P=0.1183
P=0.3971
P=0.9994
P=0.9998
P=0.9340
P=0.7750
BM cellularity (x10^8)
WT Mda5-/-
Young
Middle-aged
Aged
B
EPCR SLAM
P=0.0009
P<0.0001
P=0.0038
P<0.0001
P<0.0001
P=0.7789
P=0.9988
P>0.9999
P=0.9033
Absolute numbers
C
LSK SLAM
P=0.6304
P<0.0001
P=0.5422
P>0.9999
P=0.0633
P=0.0002
P>0.9999
P=0.7812
P<0.0001
D
LT HSCs
P=0.0153
P<0.0001
P=0.0145
P=0.9994
P<0.0001
P<0.0001
P>0.9999
P=0.5836
P<0.0001
E
Fold change
Relative to Aged WT
0.5
1.5
2.5
Aged WT
Aged Mda5-/-
P = 0.0418
P = 0.0119
P = 0.0342
P = 0.0421
P = 0.0259
AMP
ADP
ATP
CTP
GMP
UTP
Inosine
Tyrosine
Adenine
Adenosine
F
Fold change
Relative to Aged WT
1.0
1.5
P = 0.0182
P = 0.0113
P = 0.0462
Acetyl-CoA
Isocitrate
Glutamate
Succinate
Malate
Aspartate
G
MPP1
P<0.0001
P<0.0001
P>0.9999
P<0.0001
P>0.9999
P<0.0001
P=0.9298
P=0.9716
P=0.0170
Frequency in the BM
H
MPP2
P=0.0025
P<0.0001
P=0.9881
P<0.0001
P=0.8815
P<0.0001
P=0.9949
P=0.7234
P=0.9472
I
MPP3
P=0.0247
P<0.0001
P=0.9970
P=0.0002
P=0.9722
P<0.0001
P=0.9998
P>0.9999
P<0.0001
J
MPP4
P=0.0300
P<0.0001
P=0.3034
P=0.6226
P<0.0001
P>0.9999
P=0.1538
P=0.7907
P=0.9979

Supplementary Figure 2. Reduced HSC accumulation and myeloid bias in aged *Mda5*$^{-/-}$ animals. A, Bone marrow cellularity of young, middle-aged and aged WT or *Mda5*$^{-/-}$ mice. N=10 young WT, young *Mda5*$^{-/-}$, middle aged WT and middle aged *Mda5*$^{-/-}$, N=11 aged WT and 11 aged *Mda5*$^{-/-}$ biologically independent samples in *n*=3 independent experiments. Each dot represents one mouse. Data are presented as mean values ± SD. One-way ANOVA. **B-D,** Absolute numbers of EPCR SLAM (**B,** N=10 young WT and 10 young *Mda5*$^{-/-}$, N=9 middle aged WT and 9 middle aged *Mda5*$^{-/-}$, N=9 aged WT and 8 aged *Mda5*$^{-/-}$), LSK SLAM (**C,** N=7 young WT and 7 young *Mda5*$^{-/-}$, N=7 middle aged WT and 8 middle aged *Mda5*$^{-/-}$, N=12 aged WT and 8 aged *Mda5*$^{-/-}$) and LT-HSC (**D,** N=10 young WT and 10 young *Mda5*$^{-/-}$, N=7 middle aged WT and 7 middle aged *Mda5*$^{-/-}$, N=10 aged WT and 10 aged *Mda5*$^{-/-}$),) populations of young, middle-aged and aged WT or *Mda5*$^{-/-}$ mice in *n*=3 independent experiments. Each dot represents one mouse. Data are presented as mean values ± SD. One-way ANOVA. For **B**: P(young WT vs. young *Mda5*$^{-/-}$)=0.999989348534399; P(aged WT vs. aged *Mda5*$^{-/-}$)<0.000000000000001; P(middle-aged WT vs. aged WT)<0.000000000000001; P(young WT vs. aged WT)<0.000000000000001; P(young *Mda5*$^{-/-}$ vs. aged *Mda5*$^{-/-}$)=0.000858394840950. For **C**: P(aged WT vs. aged *Mda5*$^{-/-}$)=0.0000003651; P(young WT vs. aged WT)=0.0000000037; P(middle-aged *Mda5*$^{-/-}$ vs. aged *Mda5*$^{-/-}$)=0.9999909029; P(young WT vs. young *Mda5*$^{-/-}$)=0.9999999994. For **D**: P(young WT vs. young *Mda5*$^{-/-}$)=0.999999985631903; P(aged WT vs. aged *Mda5*$^{-/-}$)=0.000000003413714; P(young WT vs. middle-aged WT)=0.000049674310114; P(middle-aged WT vs. aged WT)=0.000069542318972; P(young WT vs. aged WT)<0.000000000000001. **E-F,** The fold change of intracellular nucleotide (**E,** N=9 WT and 7 *Mda5*$^{-/-}$ for AMP and ADP, N=8 WT and 9 *Mda5*$^{-/-}$ for ATP, N=4 WT and 3 *Mda5*$^{-/-}$ for CTP, N=7 WT and 4 *Mda5*$^{-/-}$ for GMP, N=5 WT and 7 *Mda5*$^{-/-}$ for UTP, N=6 WT and 4 *Mda5*$^{-/-}$ for Inosine, N=3 WT and 3 *Mda5*$^{-/-}$ for Tyrosine, N=3 WT and 2 *Mda5*$^{-/-}$ for Adenine, N=2 WT and 2 *Mda5*$^{-/-}$ for Adenosine) and TCA (**F,** N=6 WT and 6 *Mda5*$^{-/-}$ for AcetylCoA, N=7 WT and 7 *Mda5*$^{-/-}$ for Isocitrate, N=5 WT and 3 *Mda5*$^{-/-}$ for Glutamate, N=4 WT and 3 *Mda5*$^{-/-}$ for Succinate, N=6 WT and 9 *Mda5*$^{-/-}$ for Malate, N=6 WT and 8 *Mda5*$^{-/-}$ for Aspartate) related metabolite concentrations of aged WT or *Mda5*$^{-/-}$ HSCs, relative to WT control. in *n*=3 independent experiments. Data are presented as mean values ± SD. Two-tailed unpaired t-tests. **G-J,** Bone marrow frequency of MPP1 (**G**), MPP2 (**H**), MPP3 (**I**), MPP4 (**J**) of young (N=6 WT and 5 *Mda5*$^{-/-}$ for MPP1, N=7 WT and 6 *Mda5*$^{-/-}$ for MPP2, MPP3 and MPP4) middle-aged (N=10 WT and 11 *Mda5*$^{-/-}$ for MPP1, N=15 WT and 15 *Mda5*$^{-/-}$ for MPP2, N=15 WT and 16 *Mda5*$^{-/-}$ for MPP3 and MPP4) and aged (N=11 WT and 11 *Mda5*$^{-/-}$ for MPP1, N=11 WT and 10 *Mda5*$^{-/-}$ for MPP2, N=10 WT and 11 *Mda5*$^{-/-}$

for MPP3, N=11 WT and 9 *Mda5*$^{-/-}$ for MPP4) WT or *Mda5*$^{-/-}$ mice. *n*=11 biologically independent samples in *n*=3 independent experiments (mid and aged). Each dot represents one mouse. Data are presented as mean values ± SD. One-way ANOVA. For **G**: P(young WT vs. middle-aged WT)=0.999931139552702; P(middle-aged WT vs. aged WT)<0.000000000000001; P(young WT vs. aged WT)<0.000000000000001; P(young *Mda5*$^{-/-}$ vs. middle-aged *Mda5*$^{-/-}$)>0.999999999999999; P(middle-aged *Mda5*$^{-/-}$ vs. aged *Mda5*$^{-/-}$)=0.000000455083197; P(young *Mda5*$^{-/-}$ vs. aged *Mda5*$^{-/-}$)=0.000000455083197. For **H**: P(middle-aged WT vs. aged WT)=0.000007743; P(middle-aged *Mda5*$^{-/-}$ vs. aged *Mda5*$^{-/-}$)=0.000004456; P(young WT vs. aged WT)=0.000006994. For **I**: P(aged WT vs. aged *Mda5*$^{-/-}$)=0.00000505257; P(middle-aged WT vs. aged WT)=0.00000000002; P(young WT vs. aged WT)=0.00000000058. For **J**: P(young WT vs. middle-aged WT)=0.000000963448; P(young WT vs. aged WT)=0.000003494167; P(middle-aged WT vs. aged WT)=0.999999999938.

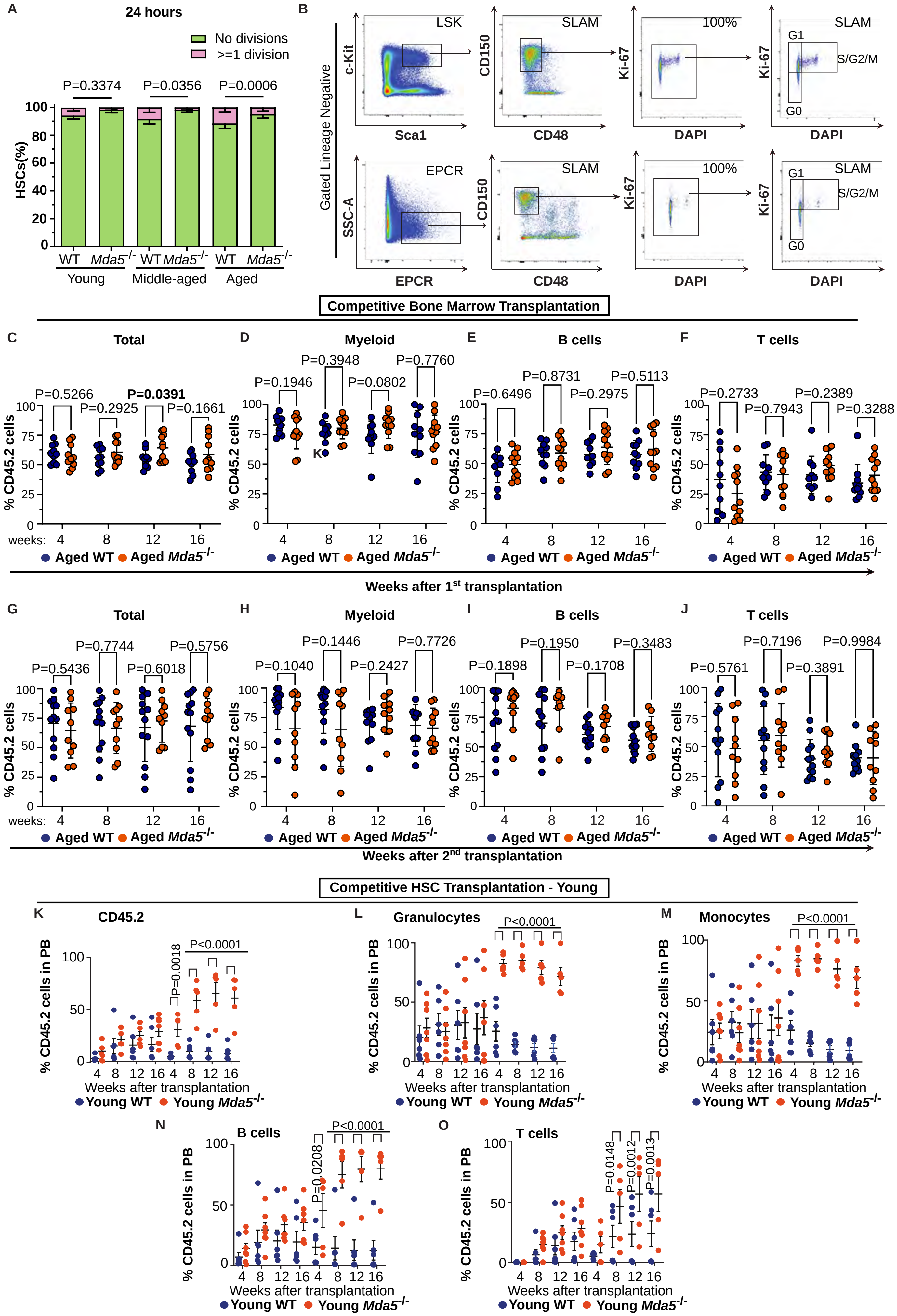
A
24 hours
No divisions
>=1 division
P=0.3374
P=0.0356
P=0.0006
HSCs(%)
WT Mda5-/- WT Mda5-/- WT Mda5-/-
Young
Middle-aged
Aged
B
Gated Lineage Negative
LSK
SLAM
100%
G1
S/G2/M
G0
c-Kit
Sca1
CD150
CD48
Ki-67
DAPI
SSC-A
EPCR
Competitive Bone Marrow Transplantation
C
Total
D
Myeloid
E
B cells
F
T cells
% CD45.2 cells
weeks:
Aged WT
Aged Mda5-/-
Weeks after 1st transplantation
P=0.5266
P=0.2925
P=0.0391
P=0.1661
P=0.1946
P=0.3948
P=0.0802
P=0.7760
P=0.6496
P=0.8731
P=0.2975
P=0.5113
P=0.2733
P=0.7943
P=0.2389
P=0.3288
G
Total
H
Myeloid
I
B cells
J
T cells
Weeks after 2nd transplantation
P=0.5436
P=0.7744
P=0.6018
P=0.5756
P=0.1040
P=0.1446
P=0.2427
P=0.7726
P=0.1898
P=0.1950
P=0.1708
P=0.3483
P=0.5761
P=0.7196
P=0.3891
P=0.9984
Competitive HSC Transplantation - Young
K
CD45.2
L
Granulocytes
M
Monocytes
N
B cells
O
T cells
% CD45.2 cells in PB
Weeks after transplantation
Young WT
Young Mda5-/-
P=0.0018
P<0.0001
P=0.0208
P=0.0148
P=0.0012
P=0.0013

Supplementary Figure 3. Aged *Mda5*$^{-/-}$ HSCs are more quiescent. A, Percentage of young (N=6 WT and 7 *Mda5*$^{-/-}$), middle-aged (N=6 WT and 7 *Mda5*$^{-/-}$), and aged (N=14 WT and 14 *Mda5*$^{-/-}$), WT or *Mda5*$^{-/-}$ HSCs that had undergone at least one division or no divisions after 24h. *n*=3 independent experiments. Data are presented as mean values SD. Two-tailed Fisher's exact test. **B,** Representative graphs depicting gating strategy for cell cycle analysis, for EPCR SLAM and LSK SLAM HSCs. **C-F,** Long-term donor hematopoietic (**C**), myeloid (**D**), B (**E**), and T (**F**) cell chimerism in the peripheral blood of recipient mice from primary competitive transplantations. N=10 WT and 11 *Mda5*$^{-/-}$ biologically independent samples in *n*=2 independent experiments. Each dot represents one mouse. Data are presented as mean values ± SD. Two-tailed unpaired t-tests. **G-J,** Long-term donor hematopoietic (**G**), myeloid (**H**), B (**I**), and T (**J**) cell chimerism in the peripheral blood of recipient mice from secondary competitive transplantations. N=12 WT and 10 *Mda5*$^{-/-}$ biologically independent samples in *n*=2 independent experiments. Each dot represents one mouse. Data are presented as mean values ± SD. Two-tailed unpaired t-tests. **K-O**, Competitive HSC transplant of young WT and *Mda5*$^{-/-}$ HSCs. Long term donor hematopoietic (**K**), granulocytic (**L**), monocytic (**M**), B-cell (**N**) and T-cell (**O**) chimerism in the peripheral blood of recipient mice from primary-HSC and secondary-Bone Marrow competitive transplantations. N=6 for young WT and N=8 young *Mda5*$^{-/-}$ recipients in primary transplantation. N=6 for young WT and N=5 young *Mda5*$^{-/-}$ recipients in secondary transplantation. Two-tailed t-tests, mean ±SD.

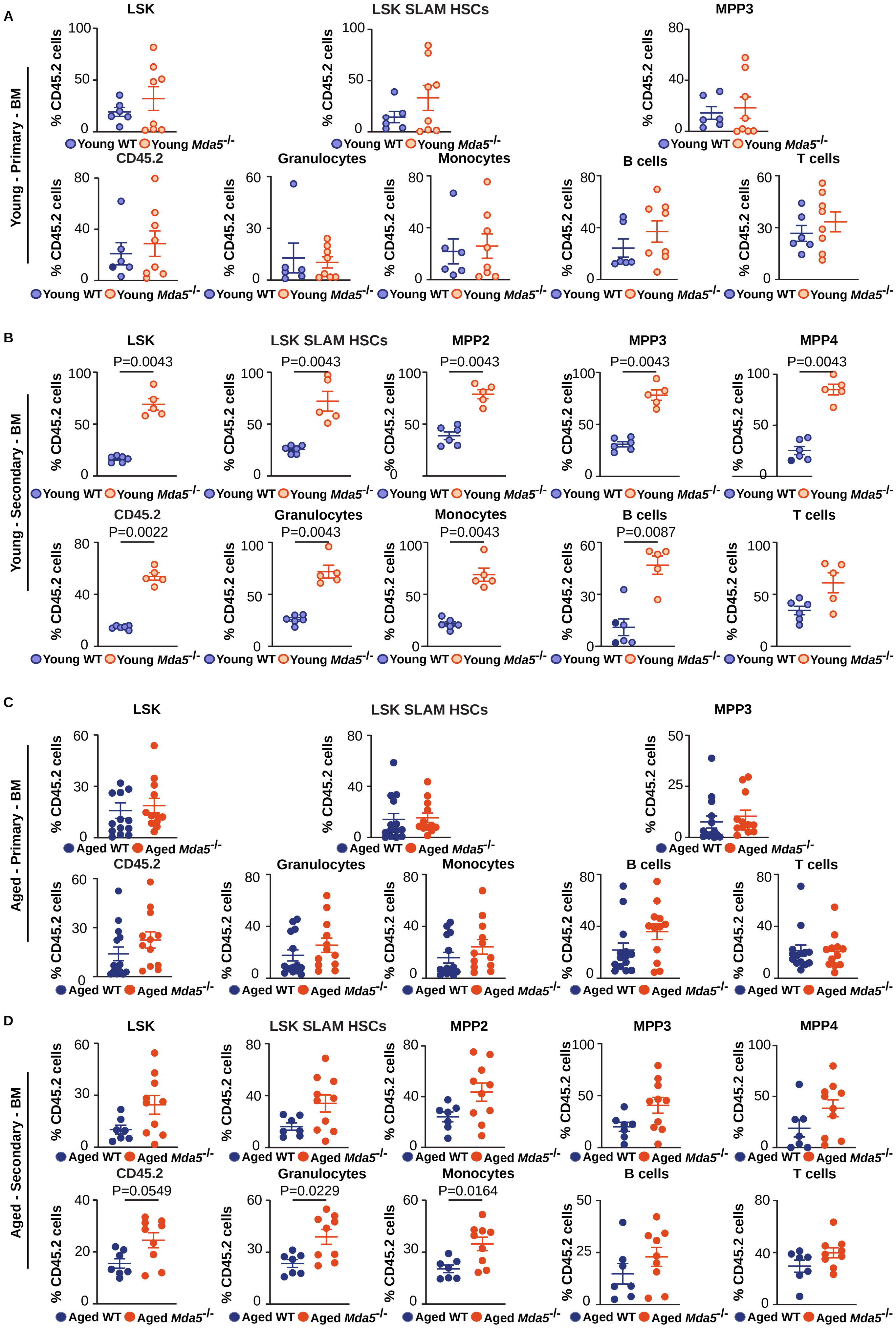
A
Young - Primary - BM
LSK
LSK SLAM HSCs
MPP3
CD45.2
Granulocytes
Monocytes
B cells
T cells
% CD45.2 cells
Young WT
Young Mda5-/-
B
Young - Secondary - BM
LSK
LSK SLAM HSCs
MPP2
MPP3
MPP4
P=0.0043
CD45.2
P=0.0022
Granulocytes
P=0.0043
Monocytes
P=0.0043
B cells
P=0.0087
T cells
% CD45.2 cells
Young WT
Young Mda5-/-
C
Aged - Primary - BM
LSK
LSK SLAM HSCs
MPP3
CD45.2
Granulocytes
Monocytes
B cells
T cells
% CD45.2 cells
Aged WT
Aged Mda5-/-
D
Aged - Secondary - BM
LSK
LSK SLAM HSCs
MPP2
MPP3
MPP4
CD45.2
P=0.0549
Granulocytes
P=0.0229
Monocytes
P=0.0164
B cells
T cells
% CD45.2 cells
Aged WT
Aged Mda5-/-

Supplementary Figure 4. Contribution of young and aged WT and *Mda5*$^{-/-}$ to the bone marrow. A-B, Competitive HSC transplant of young WT and *Mda5*$^{-/-}$ HSCs. Long term donor cell chimerism of the indicated populations in the bone marrow of recipient mice from primary-HSC (**A**) and secondary (**B**) Bone Marrow competitive transplantations. N=6 for young WT and N=8 young *Mda5*$^{-/-}$ recipients in primary transplantation. N=6 for young WT and N=5 young *Mda5*$^{-/-}$ recipients in secondary transplantation. Mann-whitney test, mean ±SD. **C-D**, Competitive HSC transplant of aged WT and *Mda5*$^{-/-}$ HSCs. Long term donor cell chimerism of the indicated population in the bone marrow of recipient mice from primary (**C**)-HSC and secondary (**D**) Bone Marrow competitive transplantations. N=14 for aged WT and N=12 aged *Mda5*$^{-/-}$ recipients in primary transplantation. N=7 for aged WT and N=10 aged *Mda5*$^{-/-}$ recipients in secondary transplantation. . Mann-whitney test, mean ±SD.

A

Ingenuity Pathway Analysis	-log(p-value)					
	WT Aged vs WT Young	*Mda5*$^{-/-}$ Aged *vs* *Mda5*$^{-/-}$ Young	WT Middle-aged vs WT Young	*Mda5*$^{-/-}$ Middle-aged *vs* *Mda5*$^{-/-}$ Young	WT Aged vs WT Middle -aged	*Mda5*$^{-/-}$ Aged vs *Mda5*$^{-/-}$ Middle-aged
Response of EIF2AK4 (GCN2) to amino acid deficiency		34.5			8.98	44.2
Eukaryotic Translation Elongation		30.3			9.72	39.5
EIF2 Signaling		21			7.34	28.8
Unfolded protein response	3.45	2.38				
Cellular response to heat stress	1.27				1.45	
Role of PKR in Interferon Induction and Antiviral Response	1.59	1.53		3	1.87	

Supplementary Figure 5. HSF1 as an upstream regulator of genes deregulated in *Mda5*$^{-/-}$ HSCs. A, Ingenuity Pathway Analysis for canonical pathways for the indicated comparisons.

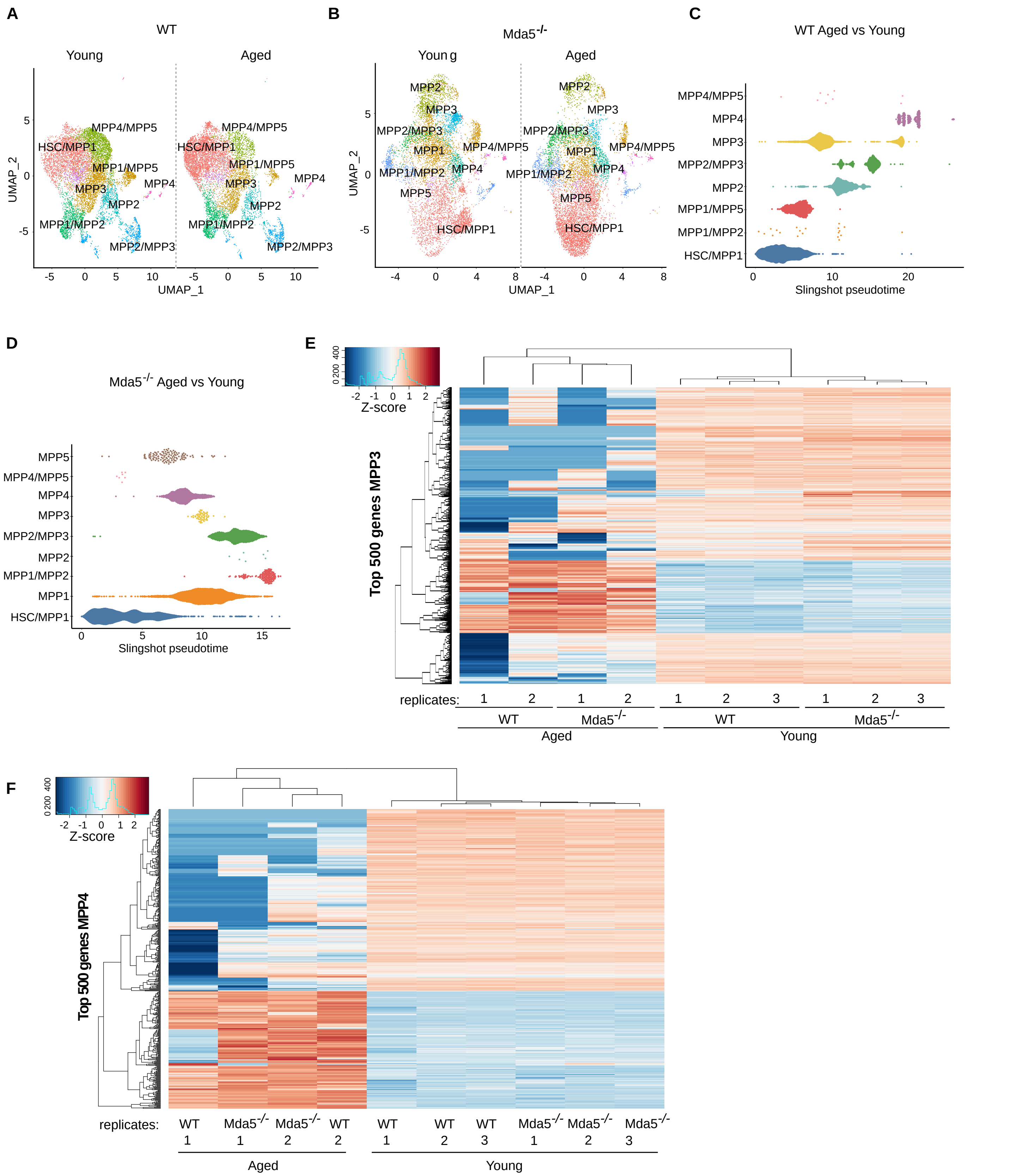

Supplementary Figure 6. Aged Mda5-/- HSCs exhibit enhanced proteostasis. A-B, UMAP projections of young and aged single-cell transcriptomes from WT (A, three young and two aged) and Mda5-/- (B, three young and two aged) donors, highlighting the different hematopoietic populations. C-D, Differentiation trajectory of cell type clusters represented in pseudotime ordering inferred by Slingshot for integrated WT (C) and Mda5-/- (D) datasets. E-F, Heatmaps of top 500 most variably expressed genes, represented as z-scores from log normalized counts per million (logCPM) detected by pseudobulk analysis on the MPP3 (E) and MPP4 (F) cluster for WT and Mda5-/- aged and young populations.

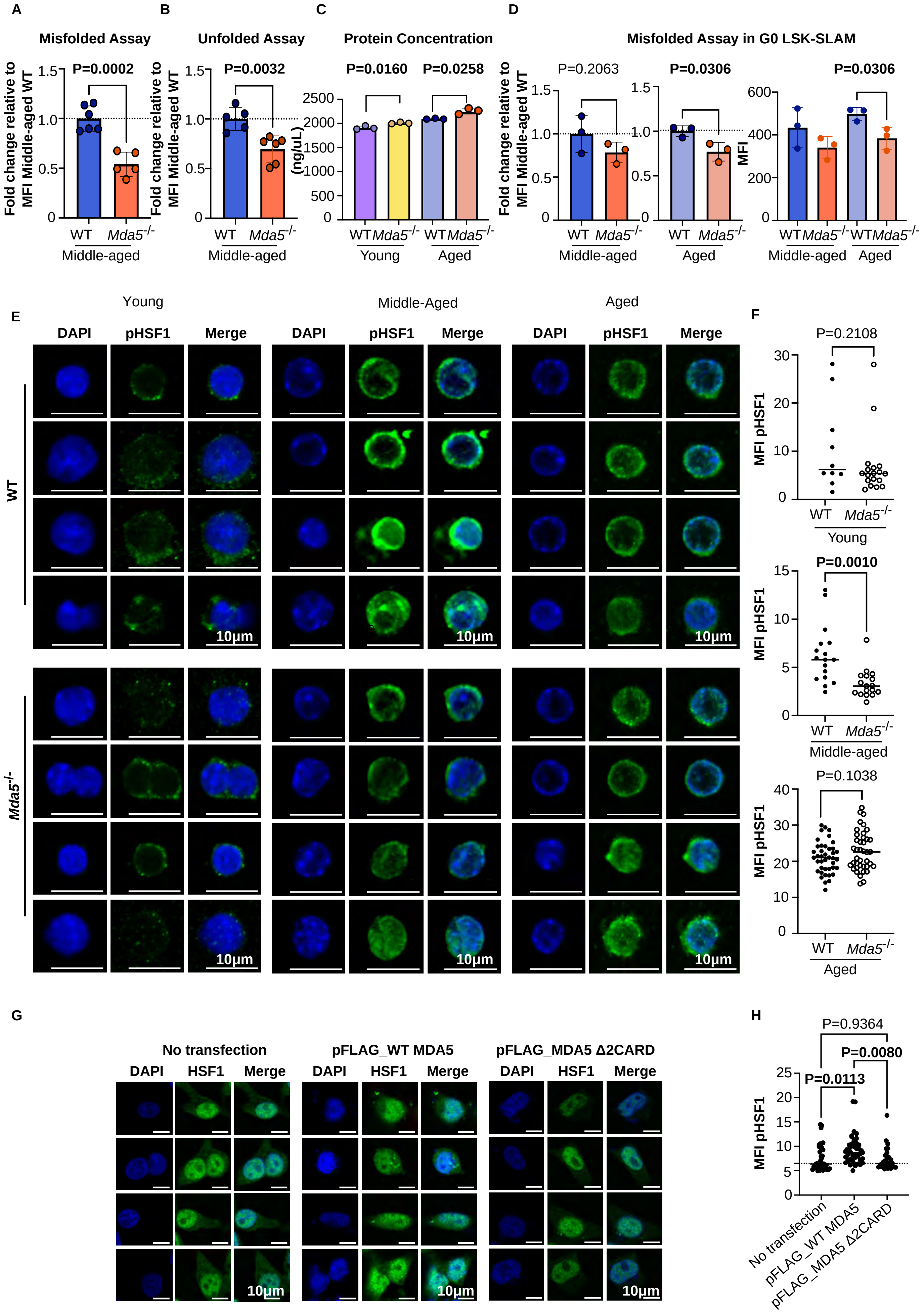
A
Misfolded Assay
P=0.0002
Fold change relative to MFI Middle-aged WT
WT Mda5-/-
Middle-aged
B
Unfolded Assay
P=0.0032
Fold change relative to MFI Middle-aged WT
WT Mda5-/-
Middle-aged
C
Protein Concentration
P=0.0160 P=0.0258
(ng/uL)
WT Mda5-/- WT Mda5-/-
Young Aged
D
Misfolded Assay in G0 LSK-SLAM
P=0.2063 P=0.0306 P=0.0306
Fold change relative to MFI Middle-aged WT
MFI
WT Mda5-/- WT Mda5-/-
Middle-aged Aged
WT Mda5-/- WT Mda5-/-
Middle-aged Aged
E
Young Middle-Aged Aged
DAPI pHSF1 Merge
WT
Mda5-/-
10μm
F
P=0.2108
MFI pHSF1
WT Mda5-/-
Young
P=0.0010
WT Mda5-/-
Middle-aged
P=0.1038
WT Mda5-/-
Aged
G
No transfection
pFLAG_WT MDA5
pFLAG_MDA5 Δ2CARD
DAPI HSF1 Merge
10μm
H
P=0.9364
P=0.0080
P=0.0113
MFI pHSF1
No transfection
pFLAG_WT MDA5
pFLAG_MDA5 Δ2CARD

Supplementary Figure 7. MDA5 overexpression partially retains HSF1 in the cytoplasm. A-B Relative accumulation of misfolded (**A,** N=6 WT and 5 *Mda5*$^{-/-}$) and unfolded (**B** N=5 WT and 6 *Mda5*$^{-/-}$) proteins in middle-aged WT or *Mda5*$^{-/-}$, normalized to the corresponding WT Mean Fluorescence Intensity (MFI). *n*=2 independent experiments. Each dot represents one mouse. Data are presented as mean values ± SD. Two-tailed unpaired t-tests. **C,** Protein concentration of 1000 sorted HSCs measured by Qubit3. *N*=3 biologically independent samples. Each dot represents one mouse. Two-tailed t-tests, mean ±SD. **D**, Relative (left) and actual (right) accumulation of misfolded proteins at G0 stage in middle-aged and aged WT or *Mda5*$^{-/-}$ HSCs, normalized to the corresponding WT Mean Fluorescence Intensity (MFI). N=3 biologically independent samples in *n*=1 experiments. Each dot represents one mouse. Two-tailed t-tests, mean ±SD. **E**, Representative immunofluorescence staining showing pHSF1 expression in young, middle age and aged WT or *Mda5*$^{-/-}$ HSCs, Scale bar 10μM. **F**, Quantification of mean Fluorescence Intensity (MFI) of pHSF1 in HSCs in young (N=10 WT and 17 *Mda5*$^{-/-}$), middle-aged (N=32 WT and 34 *Mda5*$^{-/-}$), and aged (N=37 WT and 35 *Mda5*$^{-/-}$) WT or *Mda5*$^{-/-}$ HSCs, *n*= 2 for young and 3 for middle-aged and aged independent experiments, each dot represents one cell. Two-tailed t-tests, median. **G**, Representative immunofluorescence staining showing HSF1 localization in HEK293T cells transfected with the indicated plasmids. Blue is DAPI. Scale bar 5μm. **H**, Quantification of mean Fluorescence Intensity (MFI) of HSF1 in the cytoplasm of HEK293T cells of the experiment in G (no transfection N=39, pFLAG_WT MDA5 N=32, pFLAG_MDA5Δ2CARD N=29 cells). One-way ANOVA, median.

Supplementary Table 1: Antibodies used in the study

**Antibody**	**Fluorophore**	**Company**	**Cat. Num.**	**Dilution**
CD45.2	FITC	Biolegend	109806	1:500
	Pacific Blue	Biolegend	109820	1:500
	BV605	Biolegend	109841	1:250
	PE/CY7	Biolegend	109830	1:250
CD45.1	AF700	Biolegend	110724	1:500
	PE/Cy7	Biolegend	110730	1:500
	BV605	Biolegend	110737	1:250
B220	Biotin	Biolegend	103204	1:500
	FITC	Biolegend	103206	1:500
	AF700	Biolegend	103232	1:500
	PE/Cy7	Biolegend	103222	1:500
	BV650	Biolegend	103241	1:500
Gr-1	Biotin	Biolegend	108404	1:1600
	FITC	Biolegend	108406	1:1600
	APC	Biolegend	108412	1:1000
	PE/Cy7	Biolegend	108416	1:1000
	BV650	Biolegend	108442	1:1000
	PE	Biolegend	108408	1:2000
CD11b	Biotin	Biolegend	101204	1:1600
	FITC	BD	553310	1:1600
	APC/Cy7	Biolegend	101226	1:1000
	PE/Cy7	Invitrogen	25-0112-82	1:1000
	BV650	Biolegend	101259	1:1000
	APC	Biolegend	101212	1:250
Ter119	Biotin	Biolegend	116204	1:1600
	FITC	Biolegend	116206	1:1600
	Pe/Cy7	Biolegend	116222	1:1000
	BV650	Biolegend	116235	1:1000
CD8	FITC	BD	553030	1:2000
	PE/Cy5	Biolegend	100710	1:2000
CD4	FITC	BD	553729	1:1000
	PE/Cy5	Biolegend	100410	1:1000

Sca1	PE/Cy7	Biolegend	122514	1:400
	APC/Cy7	Biolegend	108126	1:400
	APC	Biolegend	108112	1:250
C-Kit	BV421	Biolegend	105828	1:250
	BV711	Biolegend	105835	1:1000
EPCR	PE	Biolegend	141504	1:200
	APC	Biolegend	141505	1:200
CD150	PE/Dazzle	Biolegend	115936	1:400
	BV605	Biolegend	115927	1:1000
	BV421	Biolegend	115925	1:400
CD48	BV421	Biolegend	103427	1:1000
	PE/Cy7	Biolegend	103423	1:400
	APC/Cy7	Biolegend	103432	1:400
	PerCP/Cy5.5	Biolegend	103422	1:500
CD34	FITC	Biolegend	152203	1:50
CD135	PE	Biolegend	135306	1:200
	APC	Biolegend	135310	1:200
Ki-67	FITC	Biolegend	652410	1:100
	PE	Biolegend	652403	1:100
CD16/32	APC	Biolegend	101325	1:1000
	PE	Biolegend	101307	1:1000
CD127	IL-7Ra	Biolegend	135005	1:1000
	APC	Biolegend	35011	1:1000
	PE	Biolegend	135009	1:1000
NK-1.1	FITC	Biolegend	108705	1:500
CD19	FITC	Biolegend	152403	1:500
	APC/Cy7	Biolegend	115529	1:250
	BV605	Biolegend	115539	1:250
CD3e	Biotin	Biolegend	100244	1:1600

	FITC	Biolegend	100306	1:1600
	BV650	Invitrogen	416-0031-82	1:1000
	PE/Cy7	Biolegend	100319	1:1000
CD45	FITC	Biolegend	103107	1:1000
goat anti-mouse IgG, IgM (H+L)	AF488	Invitrogene	A-10680	1:2500
CD11c	FITC	Biolegend	117306	1:250
