# Supplementary material for: Lack of MDA5 delays hematopoietic aging by modulating inflammaging and proteostasis in mice
Source: Nat Commun. 2026 Feb 12;17:1645. doi: 10.1038/s41467-026-69424-x (PMC12905429; doi:10.1038/s41467-026-69424-x)
Supplement: Supplementary file 3 — Reporting Summary [file 41467_2026_69424_MOESM3_ESM.pdf]

nature portfolio

Corresponding author(s): Eirini Trompouki, Marta Derecka, Veronica Bergo

Last updated by author(s): 2025/08/01

Reporting Summary

Nature Portfolio wishes to improve the reproducibility of the work that we publish. This form provides structure for consistency and transparency in reporting. For further information on Nature Portfolio policies, see our Editorial Policies and the Editorial Policy Checklist.

Statistics

For all statistical analyses, confirm that the following items are present in the figure legend, table legend, main text, or Methods section.

n/a	Confirmed	
☐	☒	The exact sample size (*n*) for each experimental group/condition, given as a discrete number and unit of measurement
☐	☒	A statement on whether measurements were taken from distinct samples or whether the same sample was measured repeatedly
☐	☒	The statistical test(s) used AND whether they are one- or two-sided<br>*Only common tests should be described solely by name; describe more complex techniques in the Methods section.*
☒	☐	A description of all covariates tested
☒	☐	A description of any assumptions or corrections, such as tests of normality and adjustment for multiple comparisons
☐	☒	A full description of the statistical parameters including central tendency (e.g. means) or other basic estimates (e.g. regression coefficient) AND variation (e.g. standard deviation) or associated estimates of uncertainty (e.g. confidence intervals)
☐	☒	For null hypothesis testing, the test statistic (e.g. *F*, *t*, *r*) with confidence intervals, effect sizes, degrees of freedom and *P* value noted<br>*Give P values as exact values whenever suitable.*
☒	☐	For Bayesian analysis, information on the choice of priors and Markov chain Monte Carlo settings
☒	☐	For hierarchical and complex designs, identification of the appropriate level for tests and full reporting of outcomes
☒	☐	Estimates of effect sizes (e.g. Cohen's *d*, Pearson's *r*), indicating how they were calculated

Our web collection on statistics for biologists contains articles on many of the points above.

Software and code

Policy information about availability of computer code

Data collection

Sequencer: Illumina Illumina NovaSeq6000
Sequencing data demultiplexing: Illumina bcl2fastq 2.17.1
Flow cytometry: Fortessa FACS analyser, FACS ARIAIII or FACS ARIAFusion (BD Biosciences),Cytolfex
Imaging: LSM 880 (Zeiss), Axio Vert.A1, Zeiss
Cell counting: Casy Cell counter(OLS/Cytena).
qPCR: ABI StepOnePlus thermal cycler (Applied Biosystems), StepOnePlus Real-Time PCR machine (Applied Biosystems).

Data analysis

RNA-ATAC

Trim Galore
STAR aligner
Samtools
HTseq count
featureCounts
DESeq2 R package
gplots package (https://cran.r-project.org/package=gplots)
R package Shiny (https://shiny.rstudio.com/)
TEtranscript

RepeatMasker (http://www.repeatmasker.org)
MACS2
Seurat package
cellranger count
Ingenuity Pathway Analysis
Bowtie2
BEDTools
DeepTools
Wellington pyDNase package
dnase_average_profile.py
wellington_score_heatmap.py
Bowtie2 algorithm
HOMER
GSEA

For manuscripts utilizing custom algorithms or software that are central to the research but not yet described in published literature, software must be made available to editors and reviewers. We strongly encourage code deposition in a community repository (e.g. GitHub). See the Nature Portfolio guidelines for submitting code & software for further information.

Data

Policy information about availability of data

All manuscripts must include a data availability statement. This statement should provide the following information, where applicable:

- Accession codes, unique identifiers, or web links for publicly available datasets
- A description of any restrictions on data availability
- For clinical datasets or third party data, please ensure that the statement adheres to our policy

Data Availability
The BioProject PRJNA1290859 and associated SRA metadata are available at https://dataview.ncbi.nlm.nih.gov/object/PRJNA1290859?reviewer=vlqoaekcc2n8rkd0j6rhor3mbj

Research involving human participants, their data, or biological material

Policy information about studies with human participants or human data. See also policy information about sex, gender (identity/presentation), and sexual orientation and race, ethnicity and racism.

Reporting on sex and gender	n/a
Reporting on race, ethnicity, or other socially relevant groupings	n/a
Population characteristics	n/a
Recruitment	n/a
Ethics oversight	n/a

Note that full information on the approval of the study protocol must also be provided in the manuscript.

Field-specific reporting

Please select the one below that is the best fit for your research. If you are not sure, read the appropriate sections before making your selection.

☒ Life sciences ☐ Behavioural & social sciences ☐ Ecological, evolutionary & environmental sciences

For a reference copy of the document with all sections, see nature.com/documents/nr-reporting-summary-flat.pdf

Life sciences study design

All studies must disclose on these points even when the disclosure is negative.

Sample size	The sample size was determined empirically according to the nature of the experiments. Animal experiments had increased sample size in comparison to cell based experiments.
Data exclusions	One transplantation experiment was excluded due to very low animal engraftment. In the single-cell RNA-seq experiments the exclusion criteria were: low quality and doublets that were filtered out computationally
Replication	The number of replicates and independent experiments is indicated in the figure legends.

Randomization	The experiments were not randomized. Age-matched male and female littermates were used according to obtained genotype.
Blinding	For serial CFU-C experiments the investigators were blinded to group allocation during data collection and analysis. No other blinding was used since the nature of the experiments did not permit further blinding.

Reporting for specific materials, systems and methods

We require information from authors about some types of materials, experimental systems and methods used in many studies. Here, indicate whether each material, system or method listed is relevant to your study. If you are not sure if a list item applies to your research, read the appropriate section before selecting a response.

Materials & experimental systems

n/a	Involved in the study	
☐	☒	Antibodies
☐	☒	Eukaryotic cell lines
☒	☐	Palaeontology and archaeology
☐	☒	Animals and other organisms
☒	☐	Clinical data
☒	☐	Dual use research of concern
☒	☐	Plants

Methods

n/a	Involved in the study	
☒	☐	ChIP-seq
☐	☒	Flow cytometry
☒	☐	MRI-based neuroimaging

Antibodies

Antibodies used

Antibodies. The following antibodies were purchased from BioLegend and used at a dilution 1:500 dilution unless stated otherwise: anti-CD45.2/Ly5.2 (Pacific Blue or FITC, 104); anti-CD45.1/Ly5.1 (Alexa Fluor 700 or Pe/Cy7, A20); anti-CD45 (FITC, 1:1000, 30-F11); anti-CD45R/B220 (BV650 or Alexa Fluor 700 or FITC or PE/Cy7 or biotin, RA3-6B2); anti-Ly6G/Ly6C (Gr1, BV650 or PE/Cy7 or APC, 1:1000, FITC or biotin, 1:1600, RB6-8C5); anti-CD11b (BV650 or PE/Cy7 or APC/Cy7, 1:1000, FITC or biotin, 1:1600, M1/70); anti-TER119 (BV650 or PE/Cy7, 1:1000, FITC or biotin, 1:1600, TER119); anti-CD3 (BV650 or PE/Cy7, 1:1000, FITC or biotin, 1:1600, 145-2C11); anti-NK-1.1 (FITC, PK136); anti-CD19 (FITC, 1D3/CD19; anti-CD4 (PE/Cy5 or FITC, 1:1000, RM4-5); anti-CD8a (PE/Cy5 or FITC, 1:2000, 53-6.7); anti-CD117 (cKit, BV711, 1:1000, 2B8); anti-Ly-6A/E (Sca1, PE/Cy7 or APC/Cy7, 1:400, E13-161.7); anti-CD201 (EPCR, PE or APC, 1:200, RCR-16); anti-CD150 (SLAM, PE/Dazzle™ 594 or BV421, 1:400, TC15-12F12.2); anti-CD48 (PE/Cy7 or APC/Cy7, 1:400, or BV421, 1:1000, HM48-1); anti-CD34 (FITC, 1:50, SA376A4); anti-CD135/Flk2 (PE or APC, 1:200, A2F10); anti-Ki67 (FITC or PE, 1:100, 16A8); anti-CD16/32 (APC or PE, 1:1000, 93); anti-CD127 (IL-7R, APC or PE, 1:1000, A7R34); goat anti-mouse IgG, IgM (H+L) secondary (1:2500, Alexa Fluor 488, A-10680, Invitrogen).Indirect immunofluorescent analysis was conducted using primary anti-HSF1 antibodies (Enzo Life Sciences ADI-SPA-901-D, 1:500 or StressMarq SMC-118D for Rat Ab), anti-dsRNA (Sigma Aldrich MABE1134, 1:500), Phosphorylated HSF1 (Enzo Life Sciences [pSer326]HSF1 ADI-SPA-902-D 1:500) and Phosphorylated eIF2a (Cell Signalling Phospho-EIF2A (Ser51) 9721S 1:500) and secondary anti-rabbit or anti-mouse antibodies conjugated with AlexaFluor-488 dye (ThermoFisher Scientific,1:1000).

For the HSC transplanations the following antibodies were used in the respective dilutions: APC anti-mouse/human CD11b Biolegend 101212 M1/70 Rat IgG2b, κ 1/250
APC anti-mouse Ly-6A/E (Sca-1) Biolegend 108112 D7 Rat IgG2a, κ 1/250
APC/Cyanine7 anti-mouse CD19 Biolegend 115529 6D5 Rat IgG2a, κ 1/250
Brilliant Violet 421™ anti-mouse CD117 (c-Kit) Biolegend 105828 2B8 Rat IgG2b, κ 1/250
Brilliant Violet 421™ anti-mouse CD3ε Biolegend 100341 145-2C11 Armenian Hamster IgG 1/250
Brilliant Violet 605™ anti-mouse CD150 (SLAM) Biolegend 115927 TC15-12F12.2 Rat IgG2a, λ 1/1000
Brilliant Violet 605™ anti-mouse CD19 Biolegend 115539 6D5 Rat IgG2a, κ 1/250
Brilliant Violet 605™ anti-mouse CD45.1 Biolegend 110737 A20 Mouse (A.SW) IgG2a, κ 1/1000
Brilliant Violet 605™ anti-mouse CD45.2 Biolegend 109841 104 Mouse (SJL) IgG2a, κ 1/250
eBioscience™ Fixable Viability Dye eFluor™ 780 Thermofisher 65-0865-14 - - 1/2000
FITC anti-mouse CD11c Biolegend 117306 N418 Armenian Hamster IgG 1/250
FITC anti-mouse CD19 Biolegend 152404 1D3/CD19 Rat IgG2a, κ 1/250
FITC anti-mouse CD3ε Biolegend 100306 145-2C11 Armenian Hamster IgG 1/250
FITC anti-mouse CD45.2 Biolegend 109806 104 Mouse (SJL) IgG2a, κ 1/250
FITC anti-mouse Ly-6G/Ly-6C (Gr-1) Biolegend 108406 RBC6-8C5 Rat IgG2b, κ 1/500
FITC anti-mouse TER-119 Biolegend 116206 TER-119 Rat IgG2b, κ 1/250
FITC anti-mouse/human CD11b Biolegend 101206 M1/70 Rat IgG2b, κ 1/250
FITC anti-mouse/human CD45R/B220 Biolegend 103206 RA3-6B2 Rat IgG2a, κ 1/250
PE anti-mouse CD135 Biolegend 135306 A2F10 Rat IgG2a, κ 1/500
PE anti-mouse Ly-6G/Ly-6C (Gr-1) Biolegend 108408 RBC6-8C5 Rat IgG2b, κ 1/2000
PE/Cyanine7 anti-mouse CD45.2 Biolegend 109830 104 Mouse (SJL) IgG2a, κ 1/250
PerCP/Cyanine5.5 anti-mouse CD48 Biolegend 103422 HM48-1 Armenian Hamster IgG 1/500

Validation

Validation by manufacturer.APC-Cy7 anti-CD45.1/Ly5.1, Alexa Fluor 700 anti-CD45.2/Ly5.2, FITC anti-CD3e, APC or FITC anti-CD45R/B220, Pe-Cy7 anti-Sca-1, PerCP-Cy5.5 or AF-700 anti-CD48, BV-605 or PE-Cy7 or PE-Dazzle anti-CD150, PerCPCy5.5 anti Sca1, APCe780 anti c-kit, CD48-Alexa Fluor700, AF-647 anti-Ki67, – flow cytometry on mouse splenocytes
FITC or PerCP-Cy5.5 anti-CD11b/Mac-1, FITC or PerCP-Cy5.5 anti-Ly6C/Ly6G, FITC anti-Ter119, BV-421 or PE or APC-H7 or APCe780

anti-CD117/c-kit, AF-700 anti-CD34, – flow cytometry on mouse bone marrow cells
PE anti-CD135/Flk2 – flow cytometry on mouse bone marrow leukocytes
PE anti-CD201/EPCR – flow cytometry on HUVEC cells

Eukaryotic cell lines

Policy information about cell lines and Sex and Gender in Research

Cell line source(s)	HEK 293T cells
Authentication	was not performed
Mycoplasma contamination	was done with PCR
Commonly misidentified lines (See ICLAC register)	n/a

Animals and other research organisms

Policy information about studies involving animals; ARRIVE guidelines recommended for reporting animal research, and Sex and Gender in Research

Laboratory animals	Mice. All mouse experiments were carried out in accordance with the guidelines of the Federation of European Laboratory Animal Science Association and following legal approval of the Regierungspräsidium Freiburg and the Animal Care and Use Committee of the French authorities-Ciepal Azur (35-9185.81/G-18/127, 35-9185.81/G-22/058, PEA845 or apafis 2022062916507603_v5). All of the animals were maintained at the animal facility of the Max Planck Institute of Immunobiology and Epigenetics or at the IRCAN facility under specific pathogen-free conditions in individually ventilated cages with a light - dark cycle of 12 h - 12 h at 20 - 24 °C under 45 - 65% humidity. For all genotypes, gender matched female or male mice were used in the experiments and experimental and control mice were co-housed in the same facility rooms. Mice were euthanized with cervical dislocation or CO2. HSC transplantation were performed in St Jude Children's hospital. The transplantation procedure was performed according to protocols approved by the St. Jude Children's Research Hospital Institutional Animal Care and Use Committee, under animal protocol #3096.<br>Mda5-/- mice or B6.129X1(C)-Ifih1tm1.1Cln/J were purchased from Jackson Laboratory Strain #:015812. CD45.1 BL6 mice were bred in MPI-IE unless otherwise stated (Jax Strain #:002014). CD45.1 JAXBoy mice were purchased from Jackson Laboratory (C57BL/6J-Ptprcem6Lutzy/J, JAX stock #033076).
Wild animals	n/a
Reporting on sex	both males and female mice were used
Field-collected samples	n/a
Ethics oversight	All mouse experiments were carried out in accordance with the guidelines of the Federation of European Laboratory Animal Science Association and following legal approval of the Regierungspräsidium Freiburg and the Animal Care and Use Committee of the French authorities (35-9185.81/G-18/127, 35-9185.81/G-22/058, PEA871).

Note that full information on the approval of the study protocol must also be provided in the manuscript.

Plants

Seed stocks	n/a
Novel plant genotypes	n/a
Authentication	n/a

Flow Cytometry

Plots

Confirm that:

- [x] The axis labels state the marker and fluorochrome used (e.g. CD4-FITC).
- [x] The axis scales are clearly visible. Include numbers along axes only for bottom left plot of group (a 'group' is an analysis of identical markers).
- [x] All plots are contour plots with outliers or pseudocolor plots.
- [x] A numerical value for number of cells or percentage (with statistics) is provided.

Methodology

Sample preparation	Bone marrow from tibiae, femurs and hip bones were crushed in staining buffer (PBS supplemented with 2 %FBS and 1 mM EDTA) using a mortar and pestle and filtered through a 70-µm cell strainer (Flacon, 352350) to isolate the BM cells. Red cells were lysed in an Ammonium-Chloride-Potassium Buffer (ACK-Lysis Buffer, NH4Cl 150 mM, KHCO3 10 mM, EDTA 0.1 mM) for 5 min at room temperature. Cells were then washed with staining buffer.<br>For the LINE1 knockdown experiment BM was extracted from femur, pelvic bone, tibias and spine via crushing. Further preparation is described in the Methods section.
Instrument	Data were either acquired on a Fortessa FACS analyser (Becton Dickinson) or sorted using a FACS ARIA III or FACS ARIA Fusion (Becton Dickinson).
Software	FlowJo
Cell population abundance	Purity check was perfomed on a more abundant population (LSK CD48+CD150+) as the number of HSCs after sorting was on average 3,000-5,000 per animals.
Gating strategy	Unstained population and single staining controls were used to to both gating strategy and correct spectral overlaps. The gating strategy can be found in Extended Data Fig. 1 and the markers used for each population can be found in the section "Sorting strategy" in the methods section.

- [x] Tick this box to confirm that a figure exemplifying the gating strategy is provided in the Supplementary Information.
