# Supplementary material for: Lack of MDA5 delays hematopoietic aging by modulating inflammaging and proteostasis in mice
Source: Nat Commun. 2026 Feb 12;17:1645. doi: 10.1038/s41467-026-69424-x (PMC12905429; doi:10.1038/s41467-026-69424-x)
Supplement: Supplementary file 4 — Description of Supplementary Files [file 41467_2026_69424_MOESM4_ESM.docx]

**Description of Supplementary Files**

**Supplementary Data 1**: ATAC-seq in aged WT versus young *Mda5^-/-^* HSCs
**Supplementary Data 2**: ATAC-seq in aged WT versus young WT HSCs

**Supplementary Data 3**: ATAC-seq in aged WT versus aged *Mda5^-/-^* HSCs

**Supplementary Data 4**: ATAC-seq in young WT versus young *Mda5^-/-^* HSCs

**Supplementary Data 5**: Bulk RNA-seq in young, middle-aged and aged WT and *Mda5^-/-^* HSCs: genes

**Supplementary Data 6**: Bulk RNA-seq in young, middle-aged and aged WT and *Mda5^-/-^* HSCs: transposable elements

**Supplementary Data 7**: Single-cell RNAseq in HSC/MPP1

**Supplementary Data 8**: Single-cell RNAseq in MPP1

**Supplementary Data 9**: Single-cell RNAseq in MPP2

**Supplementary Data 10**: Single-cell RNAseq in MPP3

**Supplementary Data 11**: Single-cell RNAseq in MPP4

**Supplementary Data 12**: Single-cell RNAseq in MPP5
